# Nanoplatforms Targeting Intrinsically Disordered Protein Aggregation for Translational Neuroscience Applications

**DOI:** 10.3390/nano15100704

**Published:** 2025-05-08

**Authors:** Chih Hung Lo, Lenny Yi Tong Cheong, Jialiu Zeng

**Affiliations:** 1 Department of Biology, Syracuse University, Syracuse, NY 13244, USA; clo101@syr.edu; 2 Interdisciplinary Neuroscience Program, Syracuse University, Syracuse, NY 13244, USA; 3 Lee Kong Chian School of Medicine, Nanyang Technological University, Singapore 308232, Singapore; lennycheong12345@gmail.com; 4 Department of Biomedical and Chemical Engineering, Syracuse University, Syracuse, NY 13244, USA

**Keywords:** nanoplatform, intrinsically disordered protein, oligomerization, fibrillization, aggregation, nanomedicine, neuroinflammation, neurodegeneration

## Abstract

Intrinsically disordered proteins (IDPs), such as tau, beta-amyloid (Aβ), and alpha-synuclein (αSyn), are prone to misfolding, resulting in pathological aggregation and propagation that drive neurodegenerative diseases, including Alzheimer’s disease (AD), frontotemporal dementia (FTD), and Parkinson’s disease (PD). Misfolded IDPs are prone to aggregate into oligomers and fibrils, exacerbating disease progression by disrupting cellular functions in the central nervous system, triggering neuroinflammation and neurodegeneration. Furthermore, aggregated IDPs exhibit prion-like behavior, acting as seeds that are released into the extracellular space, taken up by neighboring cells, and have a propagating pathology across different regions of the brain. Conventional inhibitors, such as small molecules, peptides, and antibodies, face challenges in stability and blood–brain barrier penetration, limiting their efficacy. In recent years, nanotechnology-based strategies, such as multifunctional nanoplatforms or nanoparticles, have emerged as promising tools to address these challenges. These nanoplatforms leverage tailored designs to prevent or remodel the aggregation of IDPs and reduce associated neurotoxicity. This review discusses recent advances in nanoplatforms designed to target tau, Aβ, and αSyn aggregation, with a focus on their roles in reducing neuroinflammation and neurodegeneration. We examine critical aspects of nanoplatform design, including the choice of material backbone and targeting moieties, which influence interactions with IDPs. We also highlight key mechanisms including the interaction between nanoplatforms and IDPs to inhibit their aggregation, redirect aggregation cascade towards nontoxic, off-pathway species, and disrupt fibrillar structures into soluble forms. We further outline future directions for enhancing IDP clearance, achieving spatiotemporal control, and improving cell-specific targeting. These nanomedicine strategies offer compelling paths forward for developing more effective and targeted therapies for neurodegenerative diseases.

## 1. Introduction

Intrinsically disordered proteins (IDPs), such as tau, beta-amyloid (Aβ), and alpha-synuclein (αSyn), are characterized by the absence of a stable, well-defined tertiary structure under physiological conditions. This intrinsic flexibility enables them to participate in a wide range of essential cellular functions, including signal transduction, molecular recognition, and the formation of dynamic biomolecular condensates via liquid–liquid phase separation [1,2]. While both intrinsically disordered and folded proteins can misfold and aggregate through distinct mechanisms, the pathological misfolding and aberrant aggregation of disease-associated IDPs into toxic oligomers and fibrils remain a central feature of those involved in neurodegenerative diseases [3,4].

**Figure 1:**
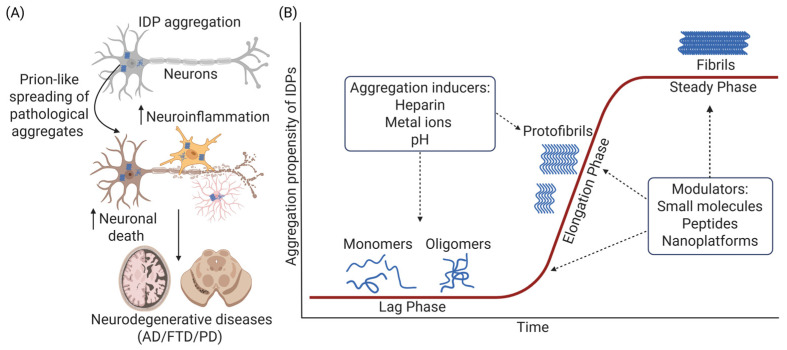
Intrinsically disordered protein (IDP) aggregation and spreading, leading to neuroinflammation and neurodegeneration. (**A**) The effect of IDP aggregation and spreading to different cell types, resulting in the propagation of the IDP pathology as well as triggering neuroinflammation and neurodegeneration in neurodegenerative diseases, including AD, FTD, and PD. (**B**) The kinetics and aggregation propensity of IDPs illustrated across different species, including monomers, oligomers, protofibrils, and fibrils under different aggregation phases such as the lag phase, elongation phase, and steady phase. Various types of aggregation inducers and modulators could remodel IDP aggregation cascade. Created with Biorender.com.

Tau, Aβ, and αSyn are notable for their ability to transition into aggregated states that disrupt cellular homeostasis, contributing to neurodegenerative diseases like Alzheimer’s disease (AD), frontotemporal dementia (FTD), and Parkinson’s disease (PD) (Figure 1A) [5]. IDPs have the propensity to template endogenous proteins and undergo the aggregation cascade through a lag phase (monomers and oligomers), an elongation phase (protofibrils), and a steady phase (fibrils) to form different aggregated species (Figure 1B). While aggregated tau forms toxic oligomers, pair helical filaments (PHFs), and neurofibrillary tangles (NFTs) [6], aggregated Aβ promotes plaque formation [7], all contributing to neuronal impairments and death in AD and FTD. αSyn aggregation forms Lewy bodies that perturb dopaminergic transmission and induce presynaptic and postsynaptic dysfunction, leading to PD pathogenesis [8]. These toxic aggregates not only compromise neuronal integrity but also trigger neuroinflammation and promote further neurotoxicity, creating a vicious cycle that accelerates disease progression [3,5,9,10,11].

As IDPs lack stable tertiary structures, they are challenging targets for therapeutic interventions [11,12]. Current strategies to modulate IDP activity mainly utilize small molecules, peptides, and antibodies designed to bind to specific motifs on these proteins and inhibit their aggregation [12,13]. However, these approaches face limitations in fully disrupting aggregation and preventing the propagation of toxic aggregates. Small molecules can interact with IDPs by stabilizing their disordered states, inhibiting interactions with other proteins, or inducing allosteric changes. Despite these strategies, achieving specificity and efficacy remains challenging due to the dynamic nature of IDPs [12,13]. Therapeutic peptides have been developed to target IDPs by mimicking natural binding partners or disrupting pathological interactions. However, their clinical application is limited by poor stability, potential immunogenicity, and difficulties in cellular delivery [14]. Monoclonal antibodies targeting IDPs aim to neutralize their toxic effects or prevent aggregation. Nevertheless, the structural flexibility of IDPs complicates antibody binding [15]. Furthermore, the complexity of the central nervous system (CNS), together with the presence of the blood–brain barrier (BBB) and blood–cerebrospinal fluid barrier, which control the entry of certain drugs and inhibitors into the brain [16,17], raises additional challenges and further limits the development of existing therapeutics.

The use of nanoplatforms as a therapeutic strategy is a forthcoming approach to targeting IDPs and preventing their aggregation and spreading within the brain [18]. Nanoparticles are solid colloidal particles with sizes in the nanometer range. Nanoparticles composed of various materials, such as polymers, metals, plasmonic metals, carbon, and lipids, not only serve as effective carriers for therapeutic agents [19,20,21,22] but also facilitate the delivery of pharmacologically active compounds across the BBB and into the brain. This is largely due to their nanoscale size and ability to modify their surfaces with targeting ligands [17,23]. Additionally, certain multimodal nanomaterials, including nanoparticles, nanotubes, nanosheets, and mesoporous structures, have been developed as nanoplatforms to deliver small molecules, small interfering RNA, and peptides for the treatment of neurodegenerative diseases [24,25,26]. Notably, some nanoplatforms incorporating plasmonic metals can chemically interact with pathological proteins while also acting as local spectroscopic probes, leveraging their unique optical properties. These dual capabilities position them as promising theranostic tools for both diagnosing and treating neurodegenerative disorders [20,27]. To achieve the goal of preventing protein aggregation and associated pathological spreading, several approaches have been proposed, including structural stabilization of the native form of amyloidogenic peptides and proteins and interfering with the assembly process of proteins and/or redirecting amyloid fibril formation toward nontoxic pathways [28,29]. A nanoplatform as a therapeutic itself can incorporate the conjugation of specific chemical moieties that either bind to IDPs and prevent oligomerization or induce conformational changes that inhibit toxic aggregation or promote nontoxic aggregation. Furthermore, these nanoplatforms can be functionalized with peptides or proteins that enhance BBB penetration and enable selective brain targeting via receptor-mediated endocytosis.

**Figure 2:**
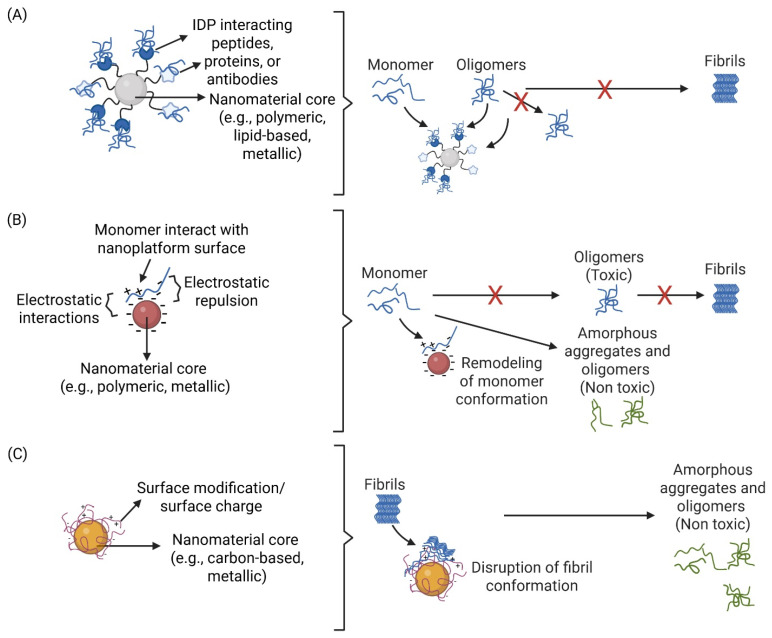
Mechanisms of action of different nanoplatforms in preventing IDP aggregation. (**A**) Nanoplatforms functionalized with IDP-interacting peptides, proteins, or antibodies bind to monomers or oligomers, preventing their progression into larger oligomers or fibrils. (**B**) Nanoplatforms interact with monomers via electrostatic interactions, inducing conformational remodeling and redirecting aggregation toward off-pathway, nontoxic oligomers. (**C**) Nanoplatforms with specific surface modifications or charges bind to fibrils, disrupting their structure and disaggregating them into amorphous aggregates or smaller oligomers. Created with Biorender.com.

In this review, we explore diverse multifunctional nanoplatform strategies targeting and sequestering pathological IDPs, including tau, Aβ, and αSyn (Figure 2), with a focus on their potential to prevent aggregation and propagation (Figure 2A), redirect aggregate formation toward nontoxic conformations and pathways (Figure 2B), and disrupt fibril formation (Figure 2C). We examine nanoplatforms designed as therapeutics to directly bind IDPs to inhibit aggregation, as well as those with intrinsic properties that modulate fibril formation. By leveraging nanomedicine approaches, these strategies hold promise for developing effective therapeutics for neurodegenerative diseases.

## 2. Tau-Targeting Nanoplatforms

In the native state, tau exists as an unfolded protein that functions as a microtubule-binding protein responsible for regulating the stability of microtubules and is essential in stabilizing the neuronal structure and intracellular cargo transport [30,31]. In the adult human brain, six tau isoforms with 352 to 441 amino acids are produced through alternative splicing of exons 2, 3, and 10. These isoforms differ by having two (2N), one (1N), or no (0N) N-terminal inserts and either four (4R) or three (3R) C-terminal microtubule-binding repeats. The adult brain expresses approximately equal amounts of 4R-tau and 3R-tau isoforms [31]. Post-translational modifications or mutations can reduce the binding ability of tau to the microtubule, leading to hyperphosphorylated tau detaching from the microtubule [32,33]. This results in an increase in the cytosolic hyperphosphorylated tau species, which becomes more vulnerable to aggregation into PHFs and NFTs [30,33]. These aggregates can lead to neuroinflammation, impaired synaptic and neuronal function, progressive neuronal loss, and increased spreading of tau pathology to neighboring cells [31,33,34].

**Figure 3:**
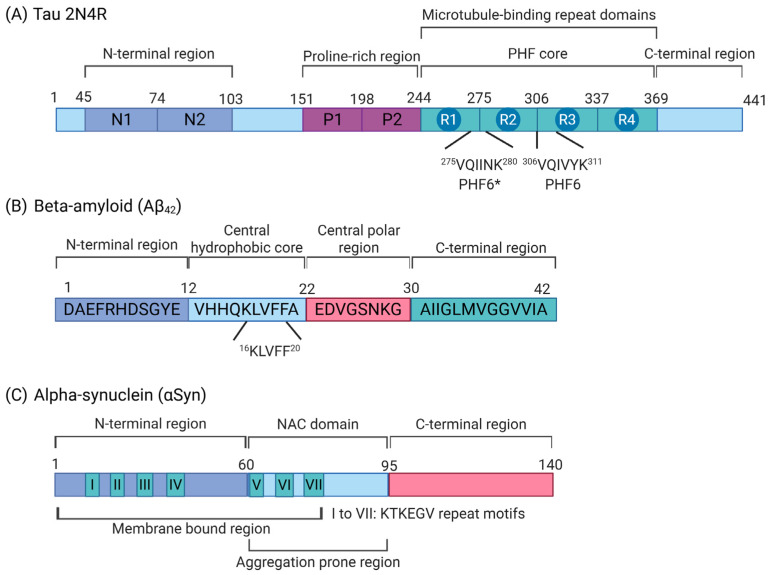
Schematic illustration of the structural features of tau (2N4R isoform), beta-amyloid (Aβ_42_), and alpha-synuclein (αSyn). (**A**) Domain organization of tau (2N4R isoform), which includes two N-terminal inserts (N1 and N2), two proline-rich regions (P1 and P2), and four microtubule-binding repeats (R1–R4). The VQIINK (PHF6*) and VQIVYK (PHF6) motifs are critical for tau aggregation. (**B**) Amino acid sequence and structural domains of Aβ_42_, consisting of an N-terminal region, a central polar region, a central hydrophobic core, and a C-terminal region. The KLVFF motif within the hydrophobic core is essential for Aβ_42_ aggregation. (**C**) The domain structure of αSyn, comprising an N-terminal region, a non-amyloid-β component (NAC) domain, and a C-terminal region. The NAC domain is the most aggregation-prone, while the N-terminal and NAC domains contain seven imperfect KTKEGV repeat motifs that can be targeted by nanoplatforms to prevent αSyn aggregation. Created with Biorender.com.

PHF6 (VQIVYK) and PHF6* (VQIINK) within the microtubule-binding domains of tau protein have been shown to be fundamental for tau aggregation and the formation of tau fibrils [33,35,36,37] (Figure 3A). A few nanoplatforms have utilized peptides consisting of the motif to bind to pathological tau and prevent their aggregation into fibrils. (D)-TLKIVW (TLK) is a peptide designed based on the atomic structure of the VQIVYK motif as a template and could bind to tau aggregates rather than tau monomers through hydrogen-bonding and hydrophobic interactions to prevent further aggregation into fibrils [38]. While it could delay tau fibril formation in vitro, its highly hydrophobic nature and low physiological stability have limited its inhibition efficacy and further therapeutic application [39]. A nanoplatform, termed as NanoTLK, is composed of a hydrophobic poly (ε-caprolactone) (PCL) core and a hydrophilic poly (ethylene glycol) (PEG) shell that self-assemble into spherical micelles. The TLK peptide is conjugated to this core–shell structure to enhance its physiological stability [40]. NanoTLK was able to reduce thioflavin-T (ThT) fluorescence when incubated with aggregated TauRD (K18, 244−372) induced by heparin and effectively reduced green punctuate in tauP301S-EGFP-transfected N2a neuronal cells compared to untreated cells. NanoTLK also did not induce any cytotoxicity to N2a cells at up to 80 μM. Furthermore, NanoTLK was shown to pass through the murine-brain microvascular endothelial cell monolayer in an in vitro BBB model, demonstrating its ability to cross the BBB, which could be attributed to the enhanced stability of NanoTLK, with a small size and enhanced transmembrane efficiency [40].

Tau-nChap is another polymeric micelle made from the self-assembly of PEG-PCL and poly (βamino ester) PAE-PCL polymers and decorated with the VQIINK peptide, which targets against the PHF6* motif of tau aggregates [41]. Similarly to the mechanism of NanoTLK, Tau-nChap bind to tau aggregates and prevents its further aggregation, leading to a reduction in ThT fluorescence after the incubation of Tau-nChap with heparin-induced aggregated full-length tau [41]. The efficacy of Tau-nChap in reducing tau aggregation was observed when the HEK293T and N2a cell lines, stably expressing GFP-Tau, had significant reductions in the number of intracellular green punctate. Tau-nChap has negligible cytotoxicity in both cell lines at up to 1000 μg mL^−1^ and reduced tau-induced toxicity. Furthermore, in an okadaic acid-induced tauopathy model of AD, there was reduced neuroinflammation, as shown by reduced TNF levels in the brain, and reduced neuronal death when treated with Tau-nChap, possibly due to a reduction in tau NFTs [41]. By the same group that developed the TLK peptide, a new peptide was designed, D-TLKIVWC (7-DP), which has been used to prevent tau aggregation. 7-DP prevents tau K18^+^ monomers from forming β-sheet structures, maintaining them in a random coil conformation. The addition of 7-DP to pre-formed tau K18^+^ fibrils reduced ThT fluorescence and led to a lower mass/charge ratio in their electrospray ionization mass spectra, suggesting that the fibrils were fragmented [42]. An MNP-DP nanoplatform, based on the conjugation of 7-DP to carboxylic acid-stabilized iron oxide magnetic nanoparticles (MNPs), retained both the preventative and fragmentation properties of 7-DP on tau aggregates. Moreover, MNP-DP did not induce cytotoxicity in N2a cells and effectively inhibited the prion-like spread of tau while also reducing tau-induced cellular toxicity. In addition, MNP-DP could cross the BBB, reduce phosphorylated tau levels, and improve memory deficits in a PS19 mouse model of AD [42]. A nanosystem comprising upconversion nanoparticles (UCNPs), leucomethylene blue (LMB), and the VQIVYK peptide, termed UCNP-LMB/VQIVYK, has shown an effect in binding to tau and preventing tau aggregation [43]. UCNP-LMB/VQIVYK effectively reduced cytotoxicity in PC12 treated with tau aggregates [43].

Protein-capped (PC) metal oxide nanoparticles, PC-Fe_3_O_4_ and PC-CdS nanoparticles, have shown an ability to inhibit their potency in inhibiting full-length tau aggregation in SH-SY5Y neuronal cells. PC-Fe_3_O_4_ and PC-CdS nanoparticles were biologically synthesized using the fungi *Fusarium oxysporum* and *Verticillium* sp., where PC-Fe_3_O_4_ nanoparticles were coated with hydrolytic proteins, while PC-CdS nanoparticles were capped with sulfate-reducing enzymes [44]. The PC-CdS and PC-Fe_3_O_4_ nanoparticles showed significant ThT fluorescence reduction (63% and 49%, respectively) [44]. In addition, the PC-CdS nanoparticles promoted the disassembly of aggregated tau fibrils, as evidenced by the presence of fragmented fibrils observed through electron microscopy. This effect is thought to result from the adsorption of tau fibrils onto agglomerated nanoparticles, which facilitates their structural disruption [44]. The PC-Fe_3_O_4_ nanoparticles exhibited no cytotoxicity in N2a cells at concentrations of up to 100 μg/mL and preserved normal cell morphology with intact neurite extensions. In contrast, the PC-CdS nanoparticles reduced cell viability by up to 50% at concentrations above 10 μg/mL, indicating dose-dependent cytotoxicity. A nanoplatform composed of nanogold and polyethylene glycol (Au-PEG) has demonstrated nanochaperone-like activity by stabilizing misfolded, aggregation-prone tau and preventing its further aggregation [45]. In a tauP301L mouse model of Alzheimer’s disease, Au-PEG treatment reduced circulating tau levels in the serum, suggesting its potential to limit tau propagation to neighboring neurons and other CNS cells. Au-PEG treatment did not induce severe toxicity and tissue damage when administered to the mouse, as validated by histological hematoxylin and eosin staining of the liver and kidneys. In addition, Au-PEG reduces the amyloidosis of AD patients’ serum samples ex vivo, as indicated by reduced ThT fluorescence [45]. Gold nanoparticles (GNPs) conjugated to β-boswellic acid (BA) (GNP-BA) inhibited the aggregation of the tau protein 1N/4R isoform in vitro. The chaperone-like feature of GNP-BA stabilized the tau monomeric structure and hence prevented its further aggregation [46].

PEGylated ceria nanoparticles (CNPs), combined with magnetic mesoporous silica nanoparticles (M-MSN), have been used to create a nanoplatform termed the tauopathy-homing nanoassembly (THN). The THN contains an anti-tau antibody, AT8, which could bind to phosphorylated tau and prevent further aggregation. In addition, the CNP component has been shown to activate autophagy by concurrently inhibiting the mammalian target of rapamycin (mTOR) and activating transcription factor EB (TFEB). FITC-labeled THN colocalizes with phosphorylated tau in autolysosomes within OA-treated SH-SY5Y, indicating that THN effectively promotes the autophagic degradation of pathogenic tau. Consequently, THN reduced tau aggregation and alleviated cognitive impairment in AD mice [47]. Unmodified poly (lactic-co-glycolic acid) (PLGA) nanoparticles have been shown to directly interact with tau and prevent their aggregation through electrostatic interactions, likely by sterically hindering tau self-association. PLGA nanoparticles also promoted an α-helical structure and reduced the β-sheet content in tau, indicating decreased conformational transition. Notably, PLGA 50:50 (50% glycolic acid, 50% lactic acid) was more effective than PLGA 75:25 (25% glycolic acid, 75% lactic acid) in preventing aggregation, suggesting that higher glycolic acid content enhances tau aggregation’s preventative effects [48]. As PLGA has been shown, in other neurodegenerative models, to increase lysosomal function and hence autophagic function [49,50], the effect of PLGA here might be partially contributed to by increased autophagic function.

## 3. Aβ-Targeting Nanoplatforms

Aβ is a ~4 kDa peptide fragment derived from the amyloid precursor protein (APP), a larger transmembrane protein widely expressed in neurons, vascular tissue, and blood cells [44]. It is produced through the sequential proteolytic cleavage of APP by β-secretase at the ectodomain and γ-secretase within the membrane [44,45]. Multiple isoforms of Aβ exist, with Aβ_40_ and Aβ_42_ being the most studied [51,52]. Among these, Aβ_42_ is particularly prone to misfolding and aggregation due to its increased hydrophobicity and two additional amino acids at the C-terminus, which enhance its neurotoxicity and tendency to form fibrils [7,53]. An imbalance between Aβ production and clearance leads to Aβ dyshomeostasis, contributing to the accumulation of misfolded species, aggregation, and extracellular plaque formation [7]. In early-stage AD, this imbalance is often driven by genetic alterations—such as APP mutations or increased β-secretase activity—resulting in Aβ overproduction. In contrast, late-onset AD is primarily associated with a breakdown of proteostasis mechanisms, impairing Aβ clearance from the brain [7]. Accumulated Aβ can also trigger neuroinflammation by activating microglial cells and initiating the NF-κB signaling cascade, ultimately contributing to neuronal dysfunction and death [7,54].

KLVFF has been shown to be an important site for Aβ aggregation and the formation of fibrils [55]; hence, peptide sequences that recognize and bind to this motif can be used in nanoplatforms to prevent further Aβ aggregation (Figure 3B). PLGA nanoparticles decorated with the LK7 peptide (LVFFARK), termed LK7@PLGA-NPs, were developed to capture and stabilize monomeric Aβ_42_ and reduce its subsequent aggregation. LK7@PLGA-NPs were nontoxic to SH-SY5Y and PC12 cells and also reduced Aβ_42_-induced cytotoxicity in both cell lines [56]. Similarly, a nanoplatform constructed based on black phosphorus (BP) nanoparticles decorated with the PEGlyated LK7 peptide, named PEG-LK7@BP, can bind to Aβ monomers to prevent its aggregation. Hence, PEG-LK7@BP reduced Aβ nucleation and amyloid elongation, redirecting the conformational transition and aggregation pathway by converting Aβ_42_ into off-pathway amorphous aggregates of nontoxic Aβ_42_ species [57]. The retro-inverso peptide RI-OR2-TAT (Ac-rGffvlkGrrrrqrrkkrGy-NH2), attached onto the surface of nanoliposomes and termed PINPs, can prevent the formation of Aβ_42_ oligomers and fibril [58]. The ‘ffvlk’ sequence of the inhibitory peptide is designed to interact with the ‘KLVFF’ sequence on the Aβ_42_ monomer. Aβ_42_ was also able interact with the positive charge of the ‘TAT’ sequence, and once captured, it inserted itself into the lipid bilayers of the PINPs. Consequently, this led to a decrease in Aβ_42_ aggregation. The attachment of retro-inverted ‘TAT’ (an HIV cell-penetrating peptide) to RI-OR2 allows PINPs to cross the BBB, potentially via increasing BBB permeability [58,59]. The application of PINPs to Tg2576 AD mice improved their cellular function and long-term recognition memory [58].

Other than using peptides, proteins with high binding affinity to Aβ species have been used in nanoplatforms to prevent Aβ aggregation. Human serum albumin (HAS)-embedded ultrasmall copper nanoclusters (CuNCs@HSA) prevent Aβ_42_ fibrillization and exhibit anti-oxidant properties. CuNCs@HSA are nontoxic to cells and could significantly decrease the levels of inflammatory cytokines TNF and IL-6 and increase the viability of Aβ_42_-treated cells [60]. Casein gold-coated nanoparticles (βCas AuNPs) were able to bind with both Aβ_42_ monomers and oligomers via nonspecific interactions, preventing Aβ_42_ aggregation. The small size of βCas AuNPs allowed for their uptake through BBB penetration in adult zebrafish, and hence restored the mobility and cognitive function of adult zebrafish exposed to Aβ_42_ [61]. βCas-coated iron oxide nanoparticles (βCas IONPs) also showed capability in preventing Aβ_42_ aggregation. βCas IONPs bind with Aβ_42_ monomers, stabilizing them and preventing their further aggregation through chaperone-like activity. βCas IONPs are nontoxic to SH-SY5Y cells, and treatment with βCas IONPs administered to zebrafish embryos and the mouse brain reduced Aβ_42_-induced toxicity [62]. Interestingly, an ICP-MS elemental analysis of iron (Fe) revealed that βCas IONPs primarily accumulated in the zebrafish brain and pancreas within 2 h of administration, with a significant decrease in pancreatic Fe levels after 24 h, suggesting clearance from the pancreas over time [62].

B6-PNi NPs made up of N-isopropylacrylamide (NiPAm) and N-tert-butylacrylamide (tBAm), a reactive oxygen species (ROS)-responsive 3-aminophenylboronic acid (APBA) moiety and a B6 peptide (CGHKAKGPRK) that allows for enhanced BBB penetration via transferrin receptor-mediated endocytosis, can prevent Aβ_42_ aggregation as well as disassemble Aβ_42_ fibrils into monomers [55]. In response to increased ROS in the AD microenvironment, B6-PNi NPs disintegrate into small nanostructures (PNi NPs) with a higher surface area, exposing more Aβ_42_ fibril binding sites. The acrylic acid group on PNi NPs can generate electrophilic carbocation to attack the nucleophilic lysine^16^ group in Aβ_16–20_, the dominant aggregation sequence of Aβ_42_, which generates a strong covalent bond between the nanostructure and Aβ_42_, thereby preventing further Aβ_42_ aggregation. Furthermore, multivalent interactions, including hydrophobic interactions, π–π stacking, and covalent attachment, between the Aβ_42_ fibril and the B6-PNi NPs can result in the disruption of the Aβ_42_ fibrillar structure [55].

The conjugation of antibodies targeting Aβ has been explored by various groups as another strategy to prevent Aβ aggregation. A nanoplatform with high affinity for the Aβ_42_ peptide was produced by coupling an anti-Aβ_42_ monoclonal antibody at the PEG chain ends of P (HDCA-co-MePEGCA) nanoparticles to form Bio-NPs. Bio-NPs bind to Aβ_42_, thereby preventing further aggregation. The application of Bio-NPs in Tg2576 AD mice improved long-term memory deficits [63]. Using a magnetic mesoporous silica nanoparticle (HA-MMSN-1F12)that contains Aβ_42_-targeting antibody 1F12 and CD44-targeting ligand hyaluronic acid (HA) to enhance BBB penetration, HA-MMSN-1F12 was shown to bind to Aβ_42_ oligomers and prevented further aggregation [64]. In APP/PS1 AD mice, HA-MMSN-1F12 showed no signs of toxicity, with normal liver and kidney function markers and no observable tissue damage in major organs. In addition, HA-MMSN-1F12 treatment resulted in reduced brain Aβ_42_ fibrils and increased soluble Aβ_42_ species, which were excreted through intestinal metabolism, thereby reducing the brain Aβ_42_ load and neuroinflammation and improving memory deficits in APP/PS1 AD mice. However, the mechanism of Aβ fibril reduction is not shown in this study [64].

Polyoxometalates (POMs) are early-transition metal–oxygen anion clusters that have been shown to bind and inhibit Aβ fibril formation, and their efficacy is size-dependent, where POMs with the biggest size show the highest amount of inhibition [65,66]. POMs bind specifically to a positively charged recognition motif (HHQK) in the 12–28 sequence of the Aβ_40_ monomer, lowering the concentration of the free monomer and shifting the equilibrium away from fibrillization. In addition, the interactions between the POM surface and Aβ_40_ oligomers could result in unfavorable conditions for nucleation and fibril growth through the blocking of direct contact between monomers [65]. Based on the properties of POMs, they have been utilized in various nanoplatforms for targeting Aβ aggregation [66,67,68,69]. One recent development is nanosized niobium POMs, Nb_10_ and TiNb_9_, which reduce S100A9 amyloid formation rate and amyloid quantity [69]. Despite the desirable properties of POM-based nanoplatforms, their interactions with Aβ are highly susceptible to environmental changes, and limited cellular infiltration poses challenges for their application in vivo, highlighting the need for more optimized designs [70].

The Shi group designed a series of nanochaperones based on mixed-shell polymeric micelles (PMs), which could prevent Aβ aggregation and accelerate clearance [71,72,73]. Recently, a nanochaperone (MSPM) based on the self-assembly of poly (β-amino ester)-*block*-poly (ε-caprolactone) (PAE-*b*-PCL) and poly (ethylene oxide)-*block*-poly (ε-caprolactone) (PEG-*b*-PCL) was formed. MSPM hydrophobic surface microdomains can bind to Aβ_42_, while hydrophilic segments act as barriers to separate Aβ_42_ particles from each other, allowing the nanochaperone to capture the Aβ_42_ peptide and subsequently prevent further Aβ_42_ aggregation. The formation of the nanochaperone–Aβ_42_ complex was susceptible to being endocytosed by microglia, which also facilitated Aβ_42_ clearance. As a result, the nanochaperone reduced the Aβ_42_ burden, attenuated Aβ_42_-induced inflammation, and rescued the cognitive deficits of APP/PS1 transgenic AD model mice [73].

Gold nanoparticles surface-functionalized with the plant-based amino acid mimosine (Mimo-AuNPs) were able to suppress spontaneous and seed-induced Aβ_42_ aggregation (~90%). Mimo-AuNPs can stabilize Aβ_42_ to keep it in its monomeric state by interacting with the hydrophobic domain of Aβ_42_ (Lys_16_ to Ala_21_), hence preventing a conformational shift towards the β-sheet structure [74]. In addition, Mimo-AuNPs were nontoxic in cultured cortical neurons and reduced Aβ_42_-mediated toxicity [74]. Near-infrared (NIR) photothermal polypyrrole nanoparticles, containing the Aβ_42_-targeting peptide LVFFA-mPEG (PEP NPs), can both prevent Aβ_42_ and disaggregate Aβ_42_ fibrils. The LVFFA peptide of the nanoparticle can bind to Aβ_42_ to prevent further aggregation [75]. PEP NPs can also interact with Aβ fibrils, leading to their disaggregation, although the exact mechanism for this is not shown. The secondary application of NIR can further induce Aβ fibril disaggregation [75]. PEP NPs are nontoxic to PC12 cells and can effectively reduce the cytotoxicity of Aβ_42_ fibrils in PC12 cells [75]. This study highlights the potential of light- and other stimuli-responsive nanoparticles to provide added benefits in treating neurodegenerative diseases, with the advantage of spatiotemporal control.

PLGA nanoparticles without any modifications were shown to interact with the hydrophobic domain of Aβ_42_, increasing the helical content and decreasing the β-sheet content, suggesting an attenuation of the conformational transition of Aβ_42_ from random coils to β-sheets, thereby preventing a conformational shift towards the β-sheet structure and thus preventing the formation of Aβ_42_ aggregates. PLGA nanoparticles did not cause any signs of toxicity in 5xFAD AD mice and effectively reduced Aβ levels [76]. In another variation, a nanocleaner [R@(ox-PLGA)-KcD] was made from a ROS-responsive PLGA core ((Polyol–ox)–PLGA), which encapsulates rapamycin, and the surface was coated with the Aβ_42_-targeting KLVFF peptide and acid-cleavable DAG peptide, which facilitates transport across the BBB [77]. The KLVFF peptide recognizes and binds to extracellular Aβ, thereby preventing further formation of Aβ aggregates. In the AD intracellular environment, with high amounts of ROS, rapamycin is rapidly released from the PLGA core of the nanocleaner, which promotes autophagy-induced Aβ degradation, thereby reducing neuroinflammation [77]. A nanosweeper composed of a cationic chitosan (CS) core coated with PEGylated-GKLVFF and Beclin-1 (TGFQGSHWIHFTANFVNT) has been shown to reduce Aβ_42_ via a similar mechanism to the nanocleaner. The KLVFF sequence can recognize and co-assemble with Aβ_42_ through hydrogen-bonding interactions [78]. The nanosweeper captures and co-assembles with extracellular Aβ, preventing the formation of Aβ aggregates. In addition, the released Beclin-1 can induce the autophagic degradation of the uptaken Aβ. The application of this nanosweeper in the brains of AD transgenic mice significantly reduced insoluble Aβ from 1539 to 914 ng/mg and soluble Aβ from 585 to 190 ng/mg, leading to improved memory function. No signs of systemic toxicity were observed, indicating good biocompatibility [78].

## 4. αSyn-Targeting Nanoplatforms

αSyn exists as an unfolded monomer in its native state and is involved in the sensing and stabilizing of curved membranes, regulating the synaptic vesicle pool, and trafficking [8,79]. αSyn consists of three distinct regions, each contributing to its specific molecular and biological roles [8]. The N-terminal region (residues 1–60) contains amphipathic repeats (KTKEGV) that tend to form α-helical structures and are essential for membrane binding [8,80,81,82]. The central portion, known as the non-amyloid-β component (NAC, residues 61–95), is the most prone to aggregation (Figure 3C) [8,80,81]. The C-terminal region (residues 96–140) carries a negative charge, is implicated in calcium ion (Ca^2+^) binding, and exhibits chaperone-like activity [8,80,81]. In pathological conditions, αSyn monomers have a tendency to self-aggregate into oligomers and insoluble fibrils, which can eventually form Lewy bodies and Lewy neurites, which have been implicated in several sporadic neurodegenerative diseases, such as PD, dementia with Lewy bodies, and multiple system atrophy [83]. Aggregated αSyn can induce dopaminergic neuronal loss, an immune response, and the spread of pathological aggregates, leading to the pathogenesis of PD [84,85].

Gold nanoparticles conjugated with β-Boswellic acid (BA) (GNP-BA), a polyphenolic compound from *Boswellia serrata*, showed an ability to prevent αSyn aggregation. The electrostatic interaction between the GNP-BA surface and αSyn monomers and/or oligomers might create unfavorable conditions for fibril growth by obstructing binding sites for the addition of new monomers, thereby preventing the aggregation of αSyn [86]. Gold nanoparticles comprising the polyphenolic compound naringenin (NAR-AuNPs) were found to interact with monomeric αSyn, forming a protein corona over the gold nanosurface. NAR-AuNPs can stabilize αSyn conformational fluctuations and hinder αSyn’s conversion into a compact cross-β-sheet conformation, which is required for subsequent fibril formation [87]. Gallic acid (GA)-conjugated polyethylenimine-coated human serum albumin nanoparticles (PEI-HSA-GA NPs) bind and stabilize αSyn monomers, thereby preventing αSyn aggregation [88]. PEI-HSA-GA NPs were nontoxic to PC-12 cells and effectively reduced αSyn-induced cell death in this neuronal model [88]. βCas IONPs have been shown to interact and capture αSyn monomers, preventing further aggregation through chaperone-like activity [62].

Nanoplatforms formed from nanocellulose (NC) and NC coated with gold atoms (NCCGA) showed an ability to adsorb to αSyn and prevent its aggregation, where NCCGA has a higher rate of adsorption to partial/full-length αSyn than NC [89]. Zinc oxide nanoparticles (ZnO NPs) are able to form strong protein–nanoparticle complexes with free αSyn monomers, hence reducing the number of free αSyn monomers available for fibrillization [90]. ZnO NPs bind to N-terminus amphipathic (KA/TKE/QGV) repeating motifs in αSyn, thereby acting as a sink to absorb free monomers and blocking aggregation sites needed for αSyn fibrillization. ZnO NPs showed no cytotoxicity in IMR32 neuroblastoma and THP-1 monocytic cells and rescued these cell lines from αSyn-induced cell death [90]. Cerium oxide nanoparticles (CeO_2_ NPs) are able to interact with Syn via electrostatic interactions, thereby increasing the duration of the lag phase and preventing αSyn elongation and aggregation. CeO_2_ NPs are nontoxic to SH-SY5Y cells and protect against neuronal cell death induced by αSyn aggregation [91]. A recent study utilizing CeO_2_ NPs showed that they prevented αSyn aggregation using the same mechanism [92]. Silver nanoparticles capped with green tea polyphenols (GTP-capped AgNPs) can modulate αSyn fibrillation by redirecting the aggregation pathway towards the formation of nontoxic, off-pathway amorphous aggregates. There was a decrease in the intensity of ThT fluorescence in samples incubated with GTP-capped AgNPs without any significant prolongation of the nucleation phase, indicative of the αSyn aggregation pathway being redirected towards the formation of amorphous aggregates [93]. Nanosheets formed from a polyphenolic compound derived from propolis (PFP) have been shown to interact and prevent αSyn aggregation. The negatively charged PFP nanosheets interact with the positively charged N-terminal of αSyn while having repulsive interactions with the negatively charged C-terminal region of αSyn, thereby redirecting αSyn aggregation toward nontoxic, off-pathway amorphous aggregates [94].

Graphene sheets and graphene quantum dots interfere with αSyn fibril formation through different mechanisms. Both disrupt fibril morphology, preventing interfilamentous assembly and resulting in aggregates of single protofilaments. Graphene oxide sheets mainly prevent αSyn aggregation by sequestering monomers and blocking nucleation and elongation [95]. In contrast, graphene quantum dots interact less strongly with monomers and fibrils, limiting secondary aggregation but not entirely preventing fibril formation [95]. In a different study, it was shown that graphene quantum dots can bind to the N-terminal cross-β part of αSyn, leading to the complete disruption of the β-sheet structure in the outer monomer. This process resulted in the release of the C-terminal region, which subsequently interacted with the opposite plane of the GQDs. This interaction disrupted the αSyn fibrillar structure, thereby reducing αSyn aggregation-induced neurotoxicity, Lewy body and Lewy neurite formation, and the spread of αSyn pathology in mice injected with αSyn pre-formed fibrils (PFFs) [96]. In addition, GQD injection reduced αSyn PFF-induced gliosis in the substantia nigra, accompanied by decreased microglia density and glial fibrillary acidic protein (GFAP) levels in astrocytes, indicative of reduced neuroinflammation [96]. GQDs also demonstrated therapeutic potential in an A53T αSyn transgenic Parkinson’s disease model, where they reduced phosphorylated αSyn levels, microglial density, and GFAP expression in astrocytes. Moreover, no loss of dopaminergic neurons, glial activation, behavioral abnormalities, or organ damage was observed following six months of prolonged GQD administration. These findings suggest that GQDs exhibit no significant long-term toxicity in vivo and are capable of being cleared from the body via urinary excretion [96]. Positively and negatively charged nanosized graphene sheets and superparamagnetic iron oxide nanoparticles (SPIONs) interact with the N- and C-terminal charged residues in αSyn, as well as the hydrophobic residues in the NAC (61–95) region. Charged nanographene sheets can interfere with both the αSyn nucleation and elongation processes, thereby preventing αSyn fibrillation. Additionally, these nanoparticles facilitate the disaggregation of mature fibrils into oligomers [97].

While many nanoplatforms, as discussed above, use peptides to bind tau and Aβ monomers or oligomers to inhibit aggregation, few have been designed to target specific aggregation motifs on αSyn for the same purpose. Various peptide-based αSyn-targeting strategies were reviewed in [98]. A recent study using a combination of in silico docking analysis and testing in vitro and in cellular models identified peptides T02 and T05 as the most effective inhibitors, with T02 binding to αSyn monomers and T05 targeting lower-order oligomers. Both peptides reduce αSyn fibril and oligomer formation in vitro and significantly suppress αSyn aggregation and cytotoxicity in yeast and human H4 cells [99]. Hence, future research could leverage these findings to design specifically targeted nanoplatforms with high efficacy in inhibiting αSyn aggregation.

## 5. Summary and Future Perspectives

IDPs such as tau, Aβ, and αSyn are highly prone to misfolding and aggregating into pathogenic species that initiate neuroinflammation, promote neuronal death, and propagate between cells, accelerating disease progression. For example, pathological tau activates microglia and astrocytes, triggering the release of proinflammatory mediators that sustain chronic neuroinflammation. As phosphorylated tau accumulates due to impaired clearance, the chronic activation of innate immune cells exacerbates neuronal damage and neurodegeneration [34]. Similarly, aggregated Aβ perpetuates neuroinflammatory signaling by persistently stimulating innate immune responses, leading to neuronal injury and cell death [100]. Both monomeric and oligomeric αSyn are known to activate microglia and induce NLRP3 inflammasome signaling [84,101]. The nanoplatforms discussed in this review mitigate IDP toxicity through three primary mechanisms: (i) binding to monomers or oligomers to prevent their aggregation into higher-order species; (ii) modulating protein conformation through electrostatic interactions, thereby redirecting aggregation toward nontoxic, off-pathway oligomers; and (iii) interacting with fibrils to disrupt their structure and disassemble them into smaller species or amorphous aggregates. While nanoplatforms employing all three strategies have demonstrated promising potential in reducing pathogenic aggregation, it is important to recognize that fibril disassembly may generate toxic intermediate species, such as soluble oligomers, which could further contribute to disease pathology [102,103,104]. Thus, careful evaluation of the bioactivity and cytotoxicity of these resulting species is essential for assessing the safety and therapeutic potential of each nanoplatform approach.

**Figure 4:**
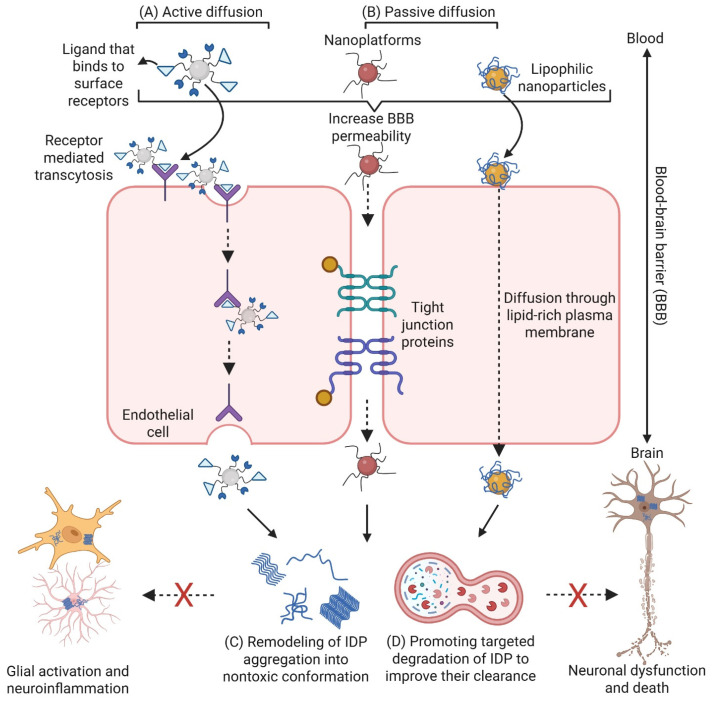
Nanoplatforms with BBB-penetrating capability can remodel toxic IDP aggregation and promote their clearance. (**A**) The active diffusion of nanoplatforms by ligand binding to the surface receptor of endothelial cells, followed by receptor-mediated transcytosis and release into the brain. (**B**) The passive diffusion of nanoplatforms, either by increased penetration through the tight function proteins or by lipid–membrane interaction-mediated diffusion into the brain. (**C**,**D**) Nanoplatforms can act through the remodeling of IDP aggregation into a nontoxic conformation (**C**) or promoting the targeted degradation of IDPs to improve their clearance (**D**), both of which contribute to attenuating glial activation and neuroinflammation, as well as rescuing neuronal dysfunction and preventing death. Created with Biorender.com.

The nanoplatforms discussed above can enter brain cells through various mechanisms. These include ligand-mediated binding to endothelial surface receptors, followed by receptor-mediated transcytosis and release into the brain parenchyma (Figure 4A); passive diffusion facilitated by the small particle size or increased BBB permeability (Figure 4B); and lipophilic interactions with the lipid-rich plasma membrane (Figure 4B). Once inside brain cells, nanoplatforms can function by remodeling IDP aggregation into nontoxic conformations (Figure 4C). The functionality of these nanoplatforms in altering IDP conformation and aggregation could be tested by biophysical and spectroscopic approaches such as cell-based fluorescence resonance energy transfer biosensors [105,106,107]. Nanoplatforms can also promote the targeted degradation of IDPs to enhance their clearance (Figure 4D). Both mechanisms help to attenuate glial activation and neuroinflammation, rescuing neuronal dysfunction and preventing cell death. Although most current nanoplatforms are evaluated in neurons and demonstrate therapeutic benefit in this cell type, it is critical to evaluate their effects in other brain cell types, such as microglia, astrocytes, and oligodendrocytes, to gain a more comprehensive understanding of their cellular responses and overall impact within the brain microenvironment.

While the nanoparticles discussed above have demonstrated potential in reducing IDP aggregation (Table 1), recent studies indicate that certain nanoparticles can instead promote their aggregation under certain conditions or cellular models [108,109]. A study investigating the effects of four nanoparticles—SiO_2_, Ti_2_O_3_, Fe_2_O_3_, and ZnO—on human H4 neuroglioma cells revealed that these nanoparticles induced alpha-synuclein inclusions in up to 60% of cells, with smaller nanoparticles showing a greater propensity to trigger inclusion formation. Mechanistically, it was shown that the abnormally high level of endogenous lysosomotropic biomolecules (e.g., sphingosine), due to impairing the integrity of endolysosomes, could be a determinant factor for the susceptibility of cells to nanoparticle-induced αSyn aggregation, and the deletion of the *GBA1* gene to increase the level of intracellular sphingosine can render cultured cells more susceptible to the formation of αSyn inclusions in response to nanoparticle treatment. These findings suggest that under conditions of lysosomal dysfunction, nanoparticles may exacerbate αSyn pathology rather than confer therapeutic benefits [109]. Therefore, nanoparticles that enhance lysosomal function may be more effective and could be integrated into a multifunctional system alongside other nanoparticles to optimize therapeutic outcomes.

The autolysosomal system is the cell’s natural degradation system, which is important for the removal of these protein aggregates [110,111]. Hence, nanoplatforms with an additional function in facilitating targeted degradation through the autolysosomal clearance of toxic protein aggregates could help increase their overall therapeutic potential [112,113,114]. For example, lysosome-targeting moieties could be incorporated into nanoplatforms to promote the autolysosomal degradation of bound or captured IDP aggregates [115,116]. Additionally, several types of nanoparticles have been developed to enhance lysosomal acidification and function [117,118], thereby improving the autophagic clearance of aggregated IDPs [119,120,121,122]. These platforms can also be conjugated with peptides or proteins that bind to IDPs to block further aggregation. Beyond lysosomal targeting, nanoplatforms can be engineered to incorporate other intracellular degradation pathways, such as the ubiquitin–proteasome system using proteolysis-targeting chimeras (PROTACs) [123,124,125] or the autophagy system using autophagy-targeting chimeras (AUTOTACs) [126,127], as well as other emerging variants [128,129], to further enhance their efficacy in clearing IDPs. The incorporation of stimuli-responsive elements, such as reactive oxygen species or near-infrared-responsive components, can further enable spatiotemporal control over IDP degradation. To ensure selective targeting, nanoplatforms may also include ligands that recognize specific brain cell types, enhancing their precision and reducing off-target effects [130]. Together, these modular design strategies enable the development of multifunctional nanoplatforms that not only inhibit and remodel toxic IDP aggregates but also promote their clearance. Such nanomedicine approaches hold promise for alleviating protein aggregation-induced neuroinflammation and neurodegeneration.

Finally, the clinical translation of nanoplatform-based therapies for IDP-associated neurodegenerative diseases requires rigorous evaluation of their biodistribution, clearance, and long-term safety [131,132]. While preliminary toxicity assessments in current studies are promising, comprehensive pharmacokinetic and pharmacodynamic profiling in clinically relevant models remains essential. The key challenges include optimizing BBB penetration, minimizing off-target effects, and ensuring metabolic clearance without systemic accumulation. Future work should prioritize standardized toxicity studies, immunogenicity assessments, and large-animal preclinical trials to address scalability and biocompatibility. By bridging these gaps, multifunctional nanoplatforms may advance from experimental tools to transformative therapies, offering hope for halting neurodegeneration driven by toxic protein aggregates [131,132].

## Figures and Tables

**Table 1:** Summary of nanoplatforms used to target tau, Aβ, and αSyn.

**Tau-Targeting Nanoplatform**			
**Nanoplatform**	**Structure**	**Mechanism of Action**	**Reference**
NanoTLK	Hydrophobic poly (ε-caprolactone) (PCL) in the core and hydrophilic poly (ethylene glycol) (PEG) in the shell	Peptide (D)-TLKIVW binds to prevent tau aggregation	[40]
Tau-nChap	PEG-PCL and poly (βamino ester) (PAE)-PCL	Peptide VQIINK binds to prevent tau aggregation	[41]
MNPs-DP	Magnetic nanoparticles (MNPs) conjugated with DP peptide	(i) Peptide D-TLKIVWC (7-DP) binds to prevent tau aggregation (ii) Changing the structure of tau fibrils	[42]
UCNPs-LMB/VQIVYK	Upconversion nanoparticles (UCNPs), leucomethylene blue (LMB), and conjugated VQIVYK peptide	(i) Peptide VQIVYK binds to prevent tau aggregation (ii) Hypochlorous acid and red light (>630 nm) on LMB yield singlet oxygen, which can disrupt Aβ aggregation	[43]
PC-Fe_3_O_4_ and PC-CdS	PC-Fe_3_O_4_ nanoparticles were coated with hydrolytic proteins, while PC-CdS nanoparticles were capped with sulfate-reducing enzymes	Adsorption of fibrils leads to disassembly of aggregated tau fibrils	[44]
Au-PEG	Nanogold and polyethylene glycol	Stabilizes misfolded and aggregation prone tau	[45]
GNP-BA	Gold nanoparticles (GNPs) conjugated to β-boswellic acid (BA)	(i) Reduced the levels of tau monomer (ii) Acted as a chaperone to stabilize the tau monomeric structure and prevent further aggregation	[46]
PLGA	Poly (lactic-co-glycolic) acid (PLGA) nanoparticles	Electrostatic interactions with tau to prevent further aggregation	[48]
**Aβ-Targeting Nanoplatform**			
**Nanoplatform**	**Structure**	**Mechanism of Action**	**Reference**
LK7@PLGA-NPs	PLGA nanoparticle with LK7 peptide (LVFFARK)	Peptide (LK7) binds to stabilize monomeric Aβ42 and prevent Aβ aggregation	[56]
PEG-LK7@BP	Black phosphorus (BP) nanoparticles coated with PEGlyated LK7 peptide (LVFFARK), an inhibitor designed to interact with Aβ aggregation motif	Peptide (LK7) binds to stabilize monomeric Aβ42 and prevent Aβ aggregation	[57]
PINPs	RI-OR2-TAT (Ac-rGffvlkGrrrrqrrkkrGy-NH2) conjugated onto liposome	RI-OR2-TAT binds to Aβ and prevents further aggregation	[58]
CuNCs@HSA	Human serum albumin (HAS)-embedded ultrasmall copper nanoclusters (CuNCs@HSA)	Binds to inhibit Aβ fibril formation	[60]
βCas AuNPs	β-Casein (β-Cas) conjugated to gold nanoparticles (AuNPs)	Nonspecific interaction with Aβ_42_ monomers and oligomers to prevent further aggregation	[61]
βCas IONPs	βCas-coated iron oxide nanoparticles (IONPs)	Nonspecific interaction with Aβ_42_ monomers and oligomers to prevent further aggregation	[62]
B6-PNi NPs	N-isopropylacrylamide (NiPAm) and N-tert-butylacrylamide (tBAm), with reactive oxygen species (ROS)-sensitive group 3-aminophenylboronic acid (APBA), conjugated with B6 peptide (CGHKAKGPRK) to allow for enhanced BBB penetration	(i) Binds to Aβ to prevent further aggregation (ii) Multivalent interactions (hydrophobic interaction, π–π stacking, covalent attachment) between Aβ fibrils and B6-PNi NPs disrupt Aβ fibrillar structure	[55]
Bio-NPs	Coupling an anti-Aβ_1-42_ monoclonal antibody to P(HDCA-co-MePEGCA) nanoparticles	Anti-Aβ antibody bind to reduce Aβ aggregation	[63]
HA-MMSN-1F12	Magnetic mesoporous silica nanoparticle (MMSN) conjugated with Aβ_42_-targeting antibody 1F12 and CD44-targeting ligand	Aβ_42_-targeting antibody 1F12 binds to Aβ_42_ oligomer and prevents aggregation	[64]
POMs	Polyoxometalate derivatives	(i) Binds to Aβ monomer to prevent Aβ aggregation (ii) Reduces nucleation and fibril growth	[66,67,68]
Nb_10_ and TiNb_9_	Nanosized niobium POMs	Reduced Aβ formation rate and quantity	[69]
Nanochaperones	Poly (β-amino ester)-*block*-poly (ε-caprolactone) (PAE-*b*-PCL) and poly (ethylene oxide)-*block*-poly (ε-caprolactone) (PEG-*b*-PCL)	Hydrophobic surface binds to Aβ to prevent Aβ aggregation, and hydrophilic surface induces steric hindrance between Aβ particles, preventing their aggregation	[73]
Mimo-AuNPs	Gold nanoparticles conjugated with plant-based amino acid mimosine ((Mimo) AuNPs)	Bind to stabilize Aβ monomer and reduce aggregation	[74]
PEP NPs	Near-infrared (NIR) photothermal polypyrrole nanoparticles coated with peptide–polyphenol complex	(i) LVFFA peptide binds to Aβ to prevent aggregation (ii) Aβ fibrils disaggregated via nanoparticle interaction upon NIR light exposure	[75]
PLGA	Poly (lactic-co-glycolic) acid (PLGA) nanoparticles in mice	Attenuation of the conformational transition of Aβ_1-42_ from random coils to β-sheets, preventing the formation and/or triggering the disassembly of Aβ aggregates	[76]
Nanocleaner	ROS-responsive PLGA core ((Polyol–ox)–PLGA), coated with KLVFF peptide and DAG peptide and encapsulating rapamycin	(i) KLVFF peptide binds to Aβ, preventing further aggregation (ii) Release of rapamycin activates autophagy to clear Aβ aggregates	[77]
Nanosweeper	Cationic chitosan (CS) core coated with PEGylated-GKLVFF, KLVFF peptide, and Beclin-1	(i) Peptide (LK7) binds to stabilize monomeric Aβ42 and prevent Aβ aggregation (ii) Release of Bectin-1 activates autophagy to clear Aβ aggregates	[78]
**αSyn-Targeting Nanoplatform**			
**Nanoplatform**	**Structure**	**Mechanism of Action**	**Reference**
GNP-BA	Gold nanoparticles conjugated with β-Boswellic acid (BA)	Electrostatic interactions obstruct binding sites for the addition of new monomers, preventing αSyn aggregation	[86]
NAR-AuNPs	Naringenin coated onto gold nanoparticles	Interacts with αSyn to prevent aggregation	[87]
PEI-HSA-GA NPs	Conjugation of gallic acid (GA) onto polyethylenimine-coated human serum albumin nanoparticles (PEI-HSA-GA NPs)	GA binds to αSyn monomer and prevents αSyn aggregation	[88]
NCCGAs	Nanocellulose (NC) and NC coated with gold atoms (NCCGAs)	Adsorb to αSyn to prevent aggregation	[89]
ZnO NP	Zinc oxide nanoparticles	Interact with αSyn to prevent aggregation	[90]
CeO_2_ NP	Cerium oxide nanoparticles	Interact with αSyn to prevent aggregation	[91,92]
GTPs-capped AgNPs	Silver nanoparticles capped with green tea polyphenols	Redirect aggregation pathway towards the formation of nontoxic, off-pathway amorphous aggregates	[93]
PFP nanosheet	Nanosheet formed from polyphenolic compound derived from propolis (PFP)	Binds and redirects αSyn aggregation toward nontoxic, off-pathway amorphous aggregates	[94]
Graphene oxide nanoparticles	Graphene oxide sheets and quantum dots	Bind αSyn monomers and oligomers to prevent αSyn aggregation	[95]
GQDs	Graphene quantum dots (GQDs)	Bind to disrupt β-sheet	[96]
Nano graphene sheets	Graphene sheets with superparamagnetic iron oxide nanoparticles (SPIONs)	(i) Charged nanographene sheets can interfere with both the nucleation and elongation processes, thereby inhibiting αSyn fibrillation (ii) Disaggregation of mature fibrils into oligomers	[97]

## References

[1] Wang B., Zhang L., Dai T., Qin Z., Lu H., Zhang L., Zhou F. (2021). Liquid–Liquid Phase Separation in Human Health and Diseases. Signal Transduct. Target. Ther..

[2] Jeon S., Jeon Y., Lim J.-Y., Kim Y., Cha B., Kim W. (2025). Emerging Regulatory Mechanisms and Functions of Biomolecular Condensates: Implications for Therapeutic Targets. Signal Transduct. Target. Ther..

[3] Utami K.H., Morimoto S., Mitsukura Y., Okano H. (2025). The Roles of Intrinsically Disordered Proteins in Neurodegeneration. Biochim. Biophys. Acta (BBA) Gen. Subj..

[4] Soto C., Pritzkow S. (2018). Protein Misfolding, Aggregation, and Conformational Strains in Neurodegenerative Diseases. Nat. Neurosci..

[5] Trivedi R., Nagarajaram H.A. (2022). Intrinsically Disordered Proteins: An Overview. Int. J. Mol. Sci..

[6] DeTure M.A., Dickson D.W. (2019). The Neuropathological Diagnosis of Alzheimer’s Disease. Mol. Neurodegener..

[7] Hampel H., Hardy J., Blennow K., Chen C., Perry G., Kim S.H., Villemagne V.L., Aisen P., Vendruscolo M., Iwatsubo T. (2021). The Amyloid-β Pathway in Alzheimer’s Disease. Mol. Psychiatry.

[8] Calabresi P., Mechelli A., Natale G., Volpicelli-Daley L., Di Lazzaro G., Ghiglieri V. (2023). Alpha-Synuclein in Parkinson’s Disease and Other Synucleinopathies: From Overt Neurodegeneration Back to Early Synaptic Dysfunction. Cell Death Dis..

[9] Lo C.H. (2022). Heterogeneous Tau Oligomers as Molecular Targets for Alzheimer’s Disease and Related Tauopathies. Biophysica.

[10] Lo C.H., Sachs J.N. (2021). The Role of Wild-Type Tau in Alzheimer’s Disease and Related Tauopathies. J Life Sci (Westlake Village).

[11] Coskuner-Weber O., Mirzanli O., Uversky V.N. (2022). Intrinsically Disordered Proteins and Proteins with Intrinsically Disordered Regions in Neurodegenerative Diseases. Biophys. Rev..

[12] Wang H., Xiong R., Lai L. (2023). Rational Drug Design Targeting Intrinsically Disordered Proteins. WIREs Comput. Mol. Sci..

[13] Tóth G., Neumann T., Berthet A., Masliah E., Spencer B., Tao J., Jobling M.F., Gardai S.J., Bertoncini C.W., Cremades N. (2019). Novel Small Molecules Targeting the Intrinsically Disordered Structural Ensemble of α-Synuclein Protect Against Diverse α-Synuclein Mediated Dysfunctions. Sci. Rep..

[14] Fantini J., Azzaz F., Di Scala C., Aulas A., Chahinian H., Yahi N. (2025). Conformationally Adaptive Therapeutic Peptides for Diseases Caused by Intrinsically Disordered Proteins (IDPs). New Paradigm for Drug Discovery: Target the Target, Not the Arrow. Pharmacol. Ther..

[15] Kouhi A., Pachipulusu V., Kapenstein T., Hu P., Epstein A.L., Khawli L.A. (2021). Brain Disposition of Antibody-Based Therapeutics: Dogma, Approaches and Perspectives. Int. J. Mol. Sci..

[16] Masoudi Asil S., Ahlawat J., Guillama Barroso G., Narayan M. (2020). Nanomaterial Based Drug Delivery Systems for the Treatment of Neurodegenerative Diseases. Biomater. Sci..

[17] Asimakidou E., Tan J.K.S., Zeng J., Lo C.H. (2024). Blood–Brain Barrier-Targeting Nanoparticles: Biomaterial Properties and Biomedical Applications in Translational Neuroscience. Pharmaceuticals.

[18] Nguyen T.T., Nguyen-Thi P.-T., Nguyen T.H.A., Ho T.-T., Tran N.-M.-A., Van Vo T., Van Vo G. (2023). Recent Advancements in Nanomaterials: A Promising Way to Manage Neurodegenerative Disorders. Mol. Diagn. Ther..

[19] Siafaka P.I., Okur N.Ü., Karantas I.D., Okur M.E., Gündoğdu E.A. (2021). Current Update on Nanoplatforms as Therapeutic and Diagnostic Tools: A Review for the Materials Used as Nanotheranostics and Imaging Modalities. Asian J. Pharm. Sci..

[20] Taverna C., Fasolato C., Brasili F., Ripanti F., Rizza C., De Marcellis A., Postorino P., Sennato S., Nucara A., Capocefalo A. (2025). Probing the Effect of the Molecular Interface of Gold Nanoparticles on the Disassembly of Insulin Amyloid Fibrils. Int. J. Biol. Macromol..

[21] Gao G., Zhang T., Zhang W., Luo Z., Zhang Z., Gu Z., Yu L., Mu Q., Sun T. (2022). High Efficiency and Related Mechanism of Au(RC) Nanoclusters on Disaggregating Aβ Fibrils. J. Colloid. Interface Sci..

[22] Liao Y.-H., Chang Y.-J., Yoshiike Y., Chang Y.-C., Chen Y.-R. (2012). Negatively Charged Gold Nanoparticles Inhibit Alzheimer’s Amyloid-β Fibrillization, Induce Fibril Dissociation, and Mitigate Neurotoxicity. Small.

[23] Jagaran K., Singh M. (2021). Nanomedicine for Neurodegenerative Disorders: Focus on Alzheimer’s and Parkinson’s Diseases. Int. J. Mol. Sci..

[24] Nayab D.E., Din F.U., Ali H., Kausar W.A., Urooj S., Zafar M., Khan I., Shabbir K., Khan G.M. (2023). Nano Biomaterials Based Strategies for Enhanced Brain Targeting in the Treatment of Neurodegenerative Diseases: An up-to-Date Perspective. J. Nanobiotechnol..

[25] Shabani L., Abbasi M., Azarnew Z., Amani A.M., Vaez A. (2023). Neuro-Nanotechnology: Diagnostic and Therapeutic Nano-Based Strategies in Applied Neuroscience. Biomed. Eng. Online.

[26] Zhu F.-D., Hu Y.-J., Yu L., Zhou X.-G., Wu J.-M., Tang Y., Qin D.-L., Fan Q.-Z., Wu A.-G. (2021). Nanoparticles: A Hope for the Treatment of Inflammation in CNS. Front. Pharmacol..

[27] Yasir M., Mishra R., Tripathi A.S., Maurya R.K., Shahi A., Zaki M.E.A., Al Hussain S.A., Masand V.H. (2024). Theranostics: A Multifaceted Approach Utilizing Nano-Biomaterials. Discov. Nano.

[28] Cohen F.E., Kelly J.W. (2003). Therapeutic Approaches to Protein-Misfolding Diseases. Nature.

[29] Bartolini M., Andrisano V. (2010). Strategies for the Inhibition of Protein Aggregation in Human Diseases. ChemBioChem.

[30] Medeiros R., Baglietto-Vargas D., LaFerla F.M. (2011). The Role of Tau in Alzheimer’s Disease and Related Disorders. CNS Neurosci. Ther..

[31] Chen Y., Yu Y. (2023). Tau and Neuroinflammation in Alzheimer’s Disease: Interplay Mechanisms and Clinical Translation. J. Neuroinflamm..

[32] Naseri N.N., Wang H., Guo J., Sharma M., Luo W. (2019). The Complexity of Tau in Alzheimer’s Disease. Neurosci. Lett..

[33] Brunello C.A., Merezhko M., Uronen R.-L., Huttunen H.J. (2020). Mechanisms of Secretion and Spreading of Pathological Tau Protein. Cell. Mol. Life Sci..

[34] Didonna A. (2020). Tau at the Interface between Neurodegeneration and Neuroinflammation. Genes Immun..

[35] Ganguly P., Do T.D., Larini L., LaPointe N.E., Sercel A.J., Shade M.F., Feinstein S.C., Bowers M.T., Shea J.-E. (2015). Tau Assembly: The Dominant Role of PHF6 (VQIVYK) in Microtubule Binding Region Repeat R3. J. Phys. Chem. B.

[36] Mirbaha H., Chen D., Morazova O.A., Ruff K.M., Sharma A.M., Liu X., Goodarzi M., Pappu R.V., Colby D.W., Mirzaei H. (2018). Inert and Seed-Competent Tau Monomers Suggest Structural Origins of Aggregation. eLife.

[37] Zeng Y., Yang J., Zhang B., Gao M., Su Z., Huang Y. (2021). The Structure and Phase of Tau: From Monomer to Amyloid Filament. Cell. Mol. Life Sci..

[38] Sievers S.A., Karanicolas J., Chang H.W., Zhao A., Jiang L., Zirafi O., Stevens J.T., Münch J., Baker D., Eisenberg D. (2011). Structure-Based Design of Non-Natural Amino-Acid Inhibitors of Amyloid Fibril Formation. Nature.

[39] Dammers C., Yolcu D., Kukuk L., Willbold D., Pickhardt M., Mandelkow E., Horn A.H.C., Sticht H., Malhis M.N., Will N. (2016). Selection and Characterization of Tau Binding ᴅ-Enantiomeric Peptides with Potential for Therapy of Alzheimer Disease. PLoS ONE.

[40] Zhu L., Xu L., Wu X., Deng F., Ma R., Liu Y., Huang F., Shi L. (2021). Tau-Targeted Multifunctional Nanoinhibitor for Alzheimer’s Disease. ACS Appl. Mater. Interfaces.

[41] Xu L., Ding Y., Ma F., Chen Y., Chen G., Zhu L., Long J., Ma R., Liu Y., Liu J. (2022). Engineering a Pathological Tau-Targeted Nanochaperone for Selective and Synergetic Inhibition of Tau Pathology in Alzheimer’s Disease. Nano Today.

[42] Hou K., Pan H., Shahpasand-Kroner H., Hu C., Abskharon R., Seidler P., Mekkittikul M., Balbirnie M., Lantz C., Sawaya M.R. (2025). D-Peptide-Magnetic Nanoparticles Fragment Tau Fibrils and Rescue Behavioral Deficits in a Mouse Model of Alzheimer’s Disease. Sci. Adv..

[43] Qiao L., Shen Y., Li G., Lv G., Li C. (2023). Hypochlorous Acid-Activated UCNPs-LMB/VQIVYK Multifunctional Nanosystem for Alzheimer’s Disease Treatment. J. Funct. Biomater..

[44] Sonawane S.K., Ahmad A., Chinnathambi S. (2019). Protein-Capped Metal Nanoparticles Inhibit Tau Aggregation in Alzheimer’s Disease. ACS Omega.

[45] Vimal S.K., Zuo H., Wang Z., Wang H., Long Z., Bhattacharyya S. (2020). Self-Therapeutic Nanoparticle That Alters Tau Protein and Ameliorates Tauopathy Toward a Functional Nanomedicine to Tackle Alzheimer’s. Small.

[46] Gharb M., Nouralishahi A., Riazi A., Riazi G. (2022). Inhibition Of Tau Protein Aggregation By a Chaperone-like β-Boswellic Acid Conjugated To Gold Nanoparticles. ACS Omega.

[47] Sun H., Zhong Y., Zhu X., Liao H., Lee J., Chen Y., Ma L., Ren J., Zhao M., Tu M. (2021). A Tauopathy-Homing and Autophagy-Activating Nanoassembly for Specific Clearance of Pathogenic Tau in Alzheimer’s Disease. ACS Nano.

[48] Paul P.S., Patel T., Cho J.-Y., Yarahmady A., Khalili A., Semenchenko V., Wille H., Kulka M., Mok S.-A., Kar S. (2024). Native PLGA Nanoparticles Attenuate Aβ-Seed Induced Tau Aggregation under in Vitro Conditions: Potential Implication in Alzheimer’s Disease Pathology. Sci. Rep..

[49] Prévot G., Soria F.N., Thiolat M.-L., Daniel J., Verlhac J.B., Blanchard-Desce M., Bezard E., Barthélémy P., Crauste-Manciet S., Dehay B. (2018). Harnessing Lysosomal PH through PLGA Nanoemulsion as a Treatment of Lysosomal-Related Neurodegenerative Diseases. Bioconjug Chem..

[50] Bourdenx M., Jonathan D., Emilie G., Federico N.S., Mireille B.-D., Erwan B., Dehay B. (2016). Nanoparticles Restore Lysosomal Acidification Defects: Implications for Parkinson and Other Lysosomal-Related Diseases. Autophagy.

[51] Sciacca M.F.M., La Rosa C., Milardi D. (2021). Amyloid-Mediated Mechanisms of Membrane Disruption. Biophysica.

[52] Wu K.Y., David D., Marco M., Chang C.A. (2022). Modeling Structural Interconversion in Alzheimers’ Amyloid Beta Peptide with Classical and Intrinsically Disordered Protein Force Fields. J. Biomol. Struct. Dyn..

[53] Chen G., Xu T., Yan Y., Zhou Y., Jiang Y., Melcher K., Xu H.E. (2017). Amyloid Beta: Structure, Biology and Structure-Based Therapeutic Development. Acta Pharmacol. Sin..

[54] Garbuz D.G., Zatsepina O.G., Evgen’ev M.B. (2021). Beta Amyloid, Tau Protein, and Neuroinflammation: An Attempt to Integrate Different Hypotheses of Alzheimer’s Disease Pathogenesis. Mol. Biol..

[55] Feng Q., Zhang X., Zhang N., Gu H., Wang N., Chen J., Yuan X., Wang L. (2024). The Dissolution, Reassembly and Further Clearance of Amyloid-β Fibrils by Tailor-Designed Dissociable Nanosystem for Alzheimer’s Disease Therapy. Exploration.

[56] Xiong N., Dong X.-Y., Zheng J., Liu F.-F., Sun Y. (2015). Design of LVFFARK and LVFFARK-Functionalized Nanoparticles for Inhibiting Amyloid β-Protein Fibrillation and Cytotoxicity. ACS Appl. Mater. Interfaces.

[57] Yang J., Liu W., Sun Y., Dong X. (2020). LVFFARK-PEG-Stabilized Black Phosphorus Nanosheets Potently Inhibit Amyloid-β Fibrillogenesis. Langmuir.

[58] Gregori M., Taylor M., Salvati E., Re F., Mancini S., Balducci C., Forloni G., Zambelli V., Sesana S., Michael M. (2017). Retro-Inverso Peptide Inhibitor Nanoparticles as Potent Inhibitors of Aggregation of the Alzheimer’s Aβ Peptide. Nanomedicine.

[59] Leibrand C.R., Paris J.J., Ghandour M.S., Knapp P.E., Kim W.-K., Hauser K.F., McRae M. (2017). HIV-1 Tat Disrupts Blood-Brain Barrier Integrity and Increases Phagocytic Perivascular Macrophages and Microglia in the Dorsal Striatum of Transgenic Mice. Neurosci. Lett..

[60] Liu L., Liu W., Sun Y., Dong X. (2024). Serum Albumin-Embedding Copper Nanoclusters Inhibit Alzheimer’s β-Amyloid Fibrillogenesis and Neuroinflammation. J. Colloid. Interface Sci..

[61] Javed I., Peng G., Xing Y., Yu T., Zhao M., Kakinen A., Faridi A., Parish C.L., Ding F., Davis T.P. (2019). Inhibition of Amyloid Beta Toxicity in Zebrafish with a Chaperone-Gold Nanoparticle Dual Strategy. Nat. Commun..

[62] Andrikopoulos N., Song Z., Wan X., Douek A.M., Javed I., Fu C., Xing Y., Xin F., Li Y., Kakinen A. (2021). Inhibition of Amyloid Aggregation and Toxicity with Janus Iron Oxide Nanoparticles. Chem. Mater..

[63] Carradori D., Balducci C., Re F., Brambilla D., Le Droumaguet B., Flores O., Gaudin A., Mura S., Forloni G., Ordoñez-Gutierrez L. (2018). Antibody-Functionalized Polymer Nanoparticle Leading to Memory Recovery in Alzheimer’s Disease-like Transgenic Mouse Model. Nanomedicine.

[64] Liu N., Liang X., Yang C., Hu S., Luo Q., Luo H. (2022). Dual-Targeted Magnetic Mesoporous Silica Nanoparticles Reduce Brain Amyloid-β Burden via Depolymerization and Intestinal Metabolism. Theranostics.

[65] Geng J., Li M., Ren J., Wang E., Qu X. (2011). Polyoxometalates as Inhibitors of the Aggregation of Amyloid β Peptides Associated with Alzheimer’s Disease. Angew. Chem. Int. Ed. Engl..

[66] Li M., Xu C., Wu L., Ren J., Wang E., Qu X. (2013). Self-Assembled Peptide–Polyoxometalate Hybrid Nanospheres: Two in One Enhances Targeted Inhibition of Amyloid β-Peptide Aggregation Associated with Alzheimer’s Disease. Small.

[67] Gao N., Dong K., Zhao A., Sun H., Wang Y., Ren J., Qu X. (2016). Polyoxometalate-Based Nanozyme: Design of a Multifunctional Enzyme for Multi-Faceted Treatment of Alzheimer’s Disease. Nano Res..

[68] Guan Y., Li M., Dong K., Gao N., Ren J., Zheng Y., Qu X. (2016). Ceria/POMs Hybrid Nanoparticles as a Mimicking Metallopeptidase for Treatment of Neurotoxicity of Amyloid-β Peptide. Biomaterials.

[69] Chaudhary H., Iashchishyn I.A., Romanova N.V., Rambaran M.A., Musteikyte G., Smirnovas V., Holmboe M., Ohlin C.A., Svedružić Ž.M., Morozova-Roche L.A. (2021). Polyoxometalates as Effective Nano-Inhibitors of Amyloid Aggregation of Pro-Inflammatory S100A9 Protein Involved in Neurodegenerative Diseases. ACS Appl. Mater. Interfaces.

[70] Ma M., Liu Z., Zhao H., Zhang H., Ren J., Qu X. (2024). Polyoxometalates: Metallodrug Agents for Combating Amyloid Aggregation. Natl. Sci. Rev..

[71] Huang F., Wang J., Qu A., Shen L., Liu J., Liu J., Zhang Z., An Y., Shi L. (2014). Maintenance of Amyloid β Peptide Homeostasis by Artificial Chaperones Based on Mixed-Shell Polymeric Micelles. Angew. Chem. Int. Ed. Engl..

[72] Huang F., Qu A., Yang H., Zhu L., Zhou H., Liu J., Long J., Shi L. (2018). Self-Assembly Molecular Chaperone to Concurrently Inhibit the Production and Aggregation of Amyloid β Peptide Associated with Alzheimer’s Disease. ACS Macro Lett..

[73] Yang H., Li X., Zhu L., Wu X., Zhang S., Huang F., Feng X., Shi L. (2019). Heat Shock Protein Inspired Nanochaperones Restore Amyloid-β Homeostasis for Preventative Therapy of Alzheimer’s Disease. Adv. Sci..

[74] Anand B.G., Wu Q., Karthivashan G., Shejale K.P., Amidian S., Wille H., Kar S. (2021). Mimosine Functionalized Gold Nanoparticles (Mimo-AuNPs) Suppress β-Amyloid Aggregation and Neuronal Toxicity. Bioact. Mater..

[75] Zhang Z., Lv M., Liu Y., Qin J., Fan Z., Du J. (2024). A Peptide-Polyphenol Coated Polypyrrole Nanoparticle for Synergetic Attenuation of Aggregation and Cytotoxicity of Amyloid-β Fibrils. Adv. Funct. Mater..

[76] Anand B., Wu Q., Nakhaei-Nejad M., Karthivashan G., Dorosh L., Amidian S., Dahal A., Li X., Stepanova M., Wille H. (2022). Significance of Native PLGA Nanoparticles in the Treatment of Alzheimer’s Disease Pathology. Bioact. Mater..

[77] Lei T., Yang Z., Xia X., Chen Y., Yang X., Xie R., Tong F., Wang X., Gao H. (2021). A Nanocleaner Specifically Penetrates the Blood–brain Barrier at Lesions to Clean Toxic Proteins and Regulate Inflammation in Alzheimer’s Disease. Acta Pharm. Sin. B.

[78] Luo Q., Lin Y.-X., Yang P.-P., Wang Y., Qi G.-B., Qiao Z.-Y., Li B.-N., Zhang K., Zhang J.-P., Wang L. (2018). A Self-Destructive Nanosweeper That Captures and Clears Amyloid β-Peptides. Nat. Commun..

[79] Gao V., Briano J.A., Komer L.E., Burré J. (2023). Functional and Pathological Effects of α-Synuclein on Synaptic SNARE Complexes. J. Mol. Biol..

[80] Nielsen M.S., Vorum H., Lindersson E., Jensen P.H. (2001). Ca2+ Binding to α-Synuclein Regulates Ligand Binding and Oligomerization. J. Biol. Chem..

[81] Fan T.-S., Liu S.C.-H., Wu R.-M. (2021). Alpha-Synuclein and Cognitive Decline in Parkinson Disease. Life.

[82] Mori A., Imai Y., Hattori N. (2020). Lipids: Key Players That Modulate α-Synuclein Toxicity and Neurodegeneration in Parkinson’s Disease. Int. J. Mol. Sci..

[83] Brás I.C., Outeiro T.F. (2021). Alpha-Synuclein: Mechanisms of Release and Pathology Progression in Synucleinopathies. Cells.

[84] Codolo G., Plotegher N., Pozzobon T., Brucale M., Tessari I., Bubacco L., de Bernard M. (2013). Triggering of Inflammasome by Aggregated α–Synuclein, an Inflammatory Response in Synucleinopathies. PLoS ONE.

[85] Grozdanov V., Bousset L., Hoffmeister M., Bliederhaeuser C., Meier C., Madiona K., Pieri L., Kiechle M., McLean P.J., Kassubek J. (2019). Increased Immune Activation by Pathologic α-Synuclein in Parkinson’s Disease. Ann. Neurol..

[86] Gharb M., Mozafari F., Arghavani P., Saboury A.A., Riazi G. (2025). Anti-Fibrillation Effect of Gold Nanoparticles Conjugated with Boswellic Acid on α-Synuclein. Res. Sq..

[87] Maity A., Mondal A., Kundu S., Shome G., Misra R., Singh A., Pal U., Mandal A.K., Bera K., Maiti N.C. (2023). Naringenin-Functionalized Gold Nanoparticles and Their Role in α-Synuclein Stabilization. Langmuir.

[88] Mohammad-Beigi H., Morshedi D., Shojaosadati S.A., Pedersen J.N., Marvian A.T., Aliakbari F., Christiansen G., Pedersen J.S., Otzen D.E. (2016). Gallic Acid Loaded onto Polyethylenimine-Coated Human Serum Albumin Nanoparticles (PEI-HSA-GA NPs) Stabilizes α-Synuclein in the Unfolded Conformation and Inhibits Aggregation. RSC Adv..

[89] Lamtar Mohammadi E., Keikha R., Outeiro T.F., Jebali A. (2024). Adsorption of Alpha-Synuclein and Inhibition of Fibril Formation by Nanocellulose and Gold-Coated-Nanocellulose. Cellulose.

[90] Asthana S., Bhattacharyya D., Kumari S., Nayak P.S., Saleem M., Bhunia A., Jha S. (2020). Interaction with Zinc Oxide Nanoparticle Kinetically Traps α-Synuclein Fibrillation into off-Pathway Non-Toxic Intermediates. Int. J. Biol. Macromol..

[91] Zand Z., Afarinesh Khaki P., Salihi A., Sharifi M., Qadir Nanakali N.M., Alasady A.A., Mohammad Aziz F., Shahpasand K., Hasan A., Falahati M. (2019). Cerium Oxide NPs Mitigate the Amyloid Formation of α-Synuclein and Associated Cytotoxicity. Int. J. Nanomed..

[92] Yao X., Guan Y., Wang J., Wang D. (2024). Cerium Oxide Nanoparticles Modulating the Parkinson’s Disease Conditions: From the Alpha Synuclein Structural Point of View and Antioxidant Properties of Cerium Oxide Nanoparticles. Heliyon.

[93] Mirzaei-Behbahani B., Meratan A.A., Moosakhani B., Mohammad-Zaheri M., Mousavi-Jarrahi Z., Nikfarjam N., Shahsavani M.B., Saboury A.A. (2024). Efficient Inhibition of Amyloid Fibrillation and Cytotoxicity of α-Synuclein and Human Insulin Using Biosynthesized Silver Nanoparticles Decorated by Green Tea Polyphenols. Sci. Rep..

[94] Rafiei Y., Salmani B., Mirzaei-Behbahani B., Taleb M., Meratan A.A., Ramezani M., Nikfarjam N., Becker S., Rezaei-Ghaleh N. (2022). Polyphenols-Based Nanosheets of Propolis Modulate Cytotoxic Amyloid Fibril Assembly of α-Synuclein. ACS Chem. Neurosci..

[95] Ghaeidamini M., Bernson D., Sasanian N., Kumar R., Esbjörner E.K. (2020). Graphene Oxide Sheets and Quantum Dots Inhibit α-Synuclein Amyloid Formation by Different Mechanisms. Nanoscale.

[96] Kim D., Yoo J.M., Hwang H., Lee J., Lee S.H., Yun S.P., Park M.J., Lee M., Choi S., Kwon S.H. (2018). Graphene Quantum Dots Prevent α-Synucleinopathy in Parkinson’s Disease. Nat. Nanotechnol..

[97] Mohammad-Beigi H., Hosseini A., Adeli M., Ejtehadi M.R., Christiansen G., Sahin C., Tu Z., Tavakol M., Dilmaghani-Marand A., Nabipour I. (2019). Mechanistic Understanding of the Interactions between Nano-Objects with Different Surface Properties and α-Synuclein. ACS Nano.

[98] Allen S.G., Meade R.M., White Stenner L.L., Mason J.M. (2023). Peptide-Based Approaches to Directly Target Alpha-Synuclein in Parkinson’s Disease. Mol. Neurodegener..

[99] Ali T.T., Merghani M., Al-Azzani M., Gatzemeier L.M., Hoppert M., Kaloyanova D., Outeiro T.F., Neumann P., Popova B., Braus G.H. (2025). Rationally Designed Peptides Inhibit the Formation of α-Synuclein Fibrils and Oligomers. Eur. J. Med. Chem..

[100] Reed-Geaghan E.G., Savage J.C., Hise A.G., Landreth G.E. (2009). CD14 and Toll-Like Receptors 2 and 4 Are Required for Fibrillar Aβ-Stimulated Microglial Activation. J. Neurosci..

[101] Scheiblich H., Bousset L., Schwartz S., Griep A., Latz E., Melki R., Heneka M.T. (2021). Microglial NLRP3 Inflammasome Activation upon TLR2 and TLR5 Ligation by Distinct α-Synuclein Assemblies. J. Immunol..

[102] Rinauro D.J., Chiti F., Vendruscolo M., Limbocker R. (2024). Misfolded Protein Oligomers: Mechanisms of Formation, Cytotoxic Effects, and Pharmacological Approaches against Protein Misfolding Diseases. Mol. Neurodegener..

[103] Cascella R., Chen S.W., Bigi A., Camino J.D., Xu C.K., Dobson C.M., Chiti F., Cremades N., Cecchi C. (2021). The Release of Toxic Oligomers from α-Synuclein Fibrils Induces Dysfunction in Neuronal Cells. Nat. Commun..

[104] Emin D., Zhang Y.P., Lobanova E., Miller A., Li X., Xia Z., Dakin H., Sideris D.I., Lam J.Y.L., Ranasinghe R.T. (2022). Small Soluble α-Synuclein Aggregates Are the Toxic Species in Parkinson’s Disease. Nat. Commun..

[105] Braun A.R., Liao E.E., Horvath M., Kalra P., Acosta K., Young M.C., Kochen N.N., Lo C.H., Brown R., Evans M.D. (2021). Potent Inhibitors of Toxic Alpha-Synuclein Identified via Cellular Time-Resolved FRET Biosensors. npj Park. Dis..

[106] Lo C.H. (2021). Recent Advances in Cellular Biosensor Technology to Investigate Tau Oligomerization. Bioeng. Transl. Med..

[107] Lo C.H., Lim C.K.-W., Ding Z., Wickramasinghe S.P., Braun A.R., Ashe K.H., Rhoades E., Thomas D.D., Sachs J.N. (2019). Targeting the Ensemble of Heterogeneous Tau Oligomers in Cells: A Novel Small Molecule Screening Platform for Tauopathies. Alzheimer’s Dement..

[108] Yuan X., Yang Y., Xia D., Meng L., He M., Liu C., Zhang Z. (2022). Silica Nanoparticles Promote α-Synuclein Aggregation and Parkinson’s Disease Pathology. Front. Neurosci..

[109] Jiang P., Gan M., Yen S.-H., Dickson D.W. (2021). Nanoparticles With Affinity for α-Synuclein Sequester α-Synuclein to Form Toxic Aggregates in Neurons With Endolysosomal Impairment. Front. Mol. Neurosci..

[110] Nixon R.A., Rubinsztein D.C. (2024). Mechanisms of Autophagy–Lysosome Dysfunction in Neurodegenerative Diseases. Nat. Rev. Mol. Cell Biol..

[111] Bonam S.R., Wang F., Muller S. (2019). Lysosomes as a Therapeutic Target. Nat. Rev. Drug Discov..

[112] Zeng J., Indajang J., Pitt D., Lo C.H. (2025). Lysosomal Acidification Impairment in Astrocyte-Mediated Neuroinflammation. J. Neuroinflamm..

[113] Quick J.D., Silva C., Wong J.H., Lim K.L., Reynolds R., Barron A.M., Zeng J., Lo C.H. (2023). Lysosomal Acidification Dysfunction in Microglia: An Emerging Pathogenic Mechanism of Neuroinflammation and Neurodegeneration. J. Neuroinflamm..

[114] Asimakidou E., Reynolds R., Barron A.M., Lo C.H. (2024). Autolysosomal Acidification Impairment as a Mediator for TNFR1 Induced Neuronal Necroptosis in Alzheimer’s Disease. Neural Regen. Res..

[115] Rathore B., Sunwoo K., Jangili P., Kim J., Kim J.H., Huang M., Xiong J., Sharma A., Yang Z., Qu J. (2019). Nanomaterial Designing Strategies Related to Cell Lysosome and Their Biomedical Applications: A Review. Biomaterials.

[116] Mondal B., Dutta T., Padhy A., Das S., Sen Gupta S. (2022). Lysosome-Targeting Strategy Using Polypeptides and Chimeric Molecules. ACS Omega.

[117] Zeng J., Acin-Perez R., Assali E.A., Martin A., Brownstein A.J., Petcherski A., Fernández-del-Rio L., Xiao R., Lo C.H., Shum M. (2023). Restoration of Lysosomal Acidification Rescues Autophagy and Metabolic Dysfunction in Non-Alcoholic Fatty Liver Disease. Nat. Commun..

[118] Lo C.H., O’Connor L.M., Loi G.W.Z., Saipuljumri E.N., Indajang J., Lopes K.M., Shirihai O.S., Grinstaff M.W., Zeng J. (2024). Acidic Nanoparticles Restore Lysosomal Acidification and Rescue Metabolic Dysfunction in Pancreatic β-Cells under Lipotoxic Conditions. ACS Nano.

[119] Cunha A., Gaubert A., Verget J., Thiolat M.-L., Barthélémy P., Latxague L., Dehay B. (2022). Trehalose-Based Nucleolipids as Nanocarriers for Autophagy Modulation: An In Vitro Study. Pharmaceutics.

[120] Arotcarena M.-L., Soria F.N., Cunha A., Doudnikoff E., Prévot G., Daniel J., Blanchard-Desce M., Barthélémy P., Bezard E., Crauste-Manciet S. (2022). Acidic Nanoparticles Protect against α-Synuclein-Induced Neurodegeneration through the Restoration of Lysosomal Function. Aging Cell.

[121] Brouillard M., Barthélémy P., Dehay B., Crauste-Manciet S., Desvergnes V. (2021). Nucleolipid Acid-Based Nanocarriers Restore Neuronal Lysosomal Acidification Defects. Front. Chem..

[122] Lo C.H., Ren M., Loi G.W.Z., Saipuljumri E.N., Indajang J., Lim K.L., Zeng J. (2024). Lysosome-Acidifying Nanoparticles Rescue A30P α-Synuclein Induced Neuronal Death in Cellular and Drosophila Models of Parkinsons Disease. bioRxiv.

[123] Békés M., Langley D.R., Crews C.M. (2022). PROTAC Targeted Protein Degraders: The Past Is Prologue. Nat. Rev. Drug Discov..

[124] Gregory J.A., Hickey C.M., Chavez J., Cacace A.M. (2024). New Therapies on the Horizon: Targeted Protein Degradation in Neuroscience. Cell Chem. Biol..

[125] Zhou Q., Wang W., Deng C. (2025). Advancements in Proteolysis Targeting Chimeras for Targeted Therapeutic Strategies in Alzheimer’s Disease. Mol. Neurobiol..

[126] Lee J., Sung K.W., Bae E.-J., Yoon D., Kim D., Lee J.S., Park D., Park D.Y., Mun S.R., Kwon S.C. (2023). Targeted Degradation of ⍺-Synuclein Aggregates in Parkinson’s Disease Using the AUTOTAC Technology. Mol. Neurodegener..

[127] Sandhof C.A., Murray H.F.B., Silva M.C., Haggarty S.J. (2024). Targeted Protein Degradation with Bifunctional Molecules as a Novel Therapeutic Modality for Alzheimer’s Disease & Beyond. Neurotherapeutics.

[128] Jiang Z., Kuo Y.-H., Arkin M.R. (2023). Autophagy Receptor-Inspired Antibody-Fusion Proteins for Targeted Intracellular Degradation. J. Am. Chem. Soc..

[129] Gong B., Zhang W., Cong W., Gu Y., Ji W., Yin T., Zhou H., Hu H., Zhuang J., Luo Y. (2024). Systemic Administration of Neurotransmitter-Derived Lipidoids-PROTACs-DNA Nanocomplex Promotes Tau Clearance and Cognitive Recovery for Alzheimer’s Disease Therapy. Adv. Healthc. Mater..

[130] Moreira R., Nóbrega C., de Almeida L.P., Mendonça L. (2024). Brain-Targeted Drug Delivery—Nanovesicles Directed to Specific Brain Cells by Brain-Targeting Ligands. J. Nanobiotechnol..

[131] Zia S., Islam Aqib A., Muneer A., Fatima M., Atta K., Kausar T., Zaheer C.-N.F., Ahmad I., Saeed M., Shafique A. (2023). Insights into Nanoparticles-Induced Neurotoxicity and Cope up Strategies. Front. Neurosci..

[132] Xuan L., Ju Z., Skonieczna M., Zhou P.-K., Huang R. (2023). Nanoparticles-Induced Potential Toxicity on Human Health: Applications, Toxicity Mechanisms, and Evaluation Models. MedComm.

